# Interconnection between Driving Style, Traffic Locus of Control, and Impulsivity in Bulgarian Drivers

**DOI:** 10.3390/bs10020058

**Published:** 2020-02-11

**Authors:** Zornitsa Totkova

**Affiliations:** Department of Psychology, Institute for Population and Human Studies, Bulgarian Academy of Sciences, 1113 Sofia, Bulgaria; z.totkova@iphs.eu

**Keywords:** driving styles, traffic locus of control, impulsivity, Bulgarian drivers, driver behaviour

## Abstract

The need for research in the field of transport psychology in Bulgaria has become more tangible in recent years, due to both the increased public intolerance to aggressive driving and the very high number of injuries and fatalities in road accidents in the country. The main objective of this study is to investigate the interconnection between Driving style, Traffic locus of control, and the Impulsivity in Bulgarian drivers. A research is conducted in order to examine the relations between the constructs in Bulgarian sample (n = 456, male = 204; female = 252; average age = 37). The results show that there are significant correlations between impulsivity and all driving styles. Positive correlations were found with the maladaptive driving styles, while the adaptive driving style was negatively associated with impulsivity. Correlations between the traffic locus of control and the driving styles were also found. These results provide a very good opportunity for further research in this area as well as for the development of prevention and training programs in the field of road safety.

## 1. Introduction

Driving is a complex process involving individual factors which are revealed in the social interaction among all road users. Considering that man is the one who drives the vehicle, an analysis of his behaviour outside the psychological science is impossible. Different perspectives in psychology explore individual differences and their influence on all aspects of the transport system [1,2]. It is logical to assume that each man’s personality is unique, which results in many variations in his behaviour, learning experience, situation evaluation, expectations, attitudes, driving style, road accidents involvement, etc. The role of the human factor in road safety, which has been studied for many decades worldwide, is of paramount importance [3]. It is clear that human factors play a crucial role in the causation of traffic accidents and researches are trying to identify and understand these factors [4]. That is why it is important to explore the patterns that identify drivers prone to risky driving behaviour and the possible negative consequences of that behaviour. Although there are many individual differences that affect the driving process, transport psychology very often focuses on personality factors. In addition, the personality approach is most often applied when considering many demographic factors, such as gender, age, etc. [5]. A number of studies in the field demonstrate that certain personality characteristics are directly related to risky driving behaviour [6,7].

### 1.1. Driving Styles and Risky Driving Behavior

The construct of driving style is an important component in effective evaluation of driving habits and behaviours [8]. In general, the driving style refers to the habitual way of driving specific for a certain driver. The most systematic viewpoint of driving style outlines eight different styles (careful, patient, risky, high-velocity, anxious, dissociative, distress-reduction, and angry styles), which are distributed in four broad domains: reckless and careless, anxious, angry and hostile, and patient and careful [9,10,11]. Many studies prove the connection between driving styles and certain driving behaviours and outcomes [9,12,13,14]. The three maladaptive domains are positively associated with frequency of reckless driving, proneness to reckless driving, rate of risky events, drunk driving, and involvement in car accidents [8,9,14,15,16]. Regarding the adaptive domain, the results reveal that drivers who have high scores in patient and careful driving styles show lower frequency of reckless driving, proneness to reckless driving, and involvement in car accidents [9,14].

There is also research data showing that the driving domains and the different styles are associated with certain personality characteristics. For instance, there is a positive correlation between the impulsive sensation-seeking personality trait and the risky, angry, and dissociative driving styles and a negative correlation with a careful driving style [17]. Regarding the Big Five, there are also studies showing a relation between the styles and personality traits. Drivers with higher levels of extroversion and lower levels of agreeableness and consciousness more often demonstrate reckless and angry driving styles; drivers with lower consciousness and higher neuroticism perform anxious style more often; the careful driving style is associated with higher levels of agreeableness, consciousness, and openness [18]. Another study reveals a positive relationship between risky, angry, high-velocity, careful, and anxious styles, and extraversion and neuroticism and a negative correlation with conscientiousness and agreeableness [8]. Extraversion is also significantly and negatively related to dissociative and anxious driving styles [9]. Research shows that positive self-esteem leads to a higher tendency of adoption careful and patient driving style, while negative self-esteem leads to a tendency of adoption dissociative or risky driving styles. The need for control is also studied: the higher the need for control, the higher the tendency to adopt angry or careful driving styles [9].

### 1.2. Personality Characteristics and Risky Driving Behavior

Although there are numerous definitions of personality, most of them share the understanding that it includes sustainable patterns of thoughts, feelings, and actions that emerge at a certain level of stability in time and context. Traffic psychology most often relies on the personality approach in which the main focus is on personality characteristics, defining the overall personality. As a result, it is believed that certain traits are inherently more dangerous than others in terms of the road environment. A person who owns more of these characteristics carries a greater risk to themselves and other road users. However, some characteristics may have a much greater impact on a person’s driving behaviour than others. Individuals with “dangerous” traits revealed to a great extent are the most problematic factor related to road safety [5].

A large number of studies attempt to connect the personality with the negative consequences of driving, particularly with the road accidents involvement, which will worsen the quality of life [19] and lead to post-traumatic stress disorder [20]. Worldwide research shows that certain personality traits are strong predictors of reckless driving and participation in road accidents. In particular, these traits are associated with driving behaviour such as speeding, traffic violation, and road accident incidents [21,22,23,24,25,26]. The in-depth analysis demonstrates that the personality characteristics most commonly associated with such behaviour are emotional stability, anxiety, aggression, anger, locus of control, sensation seeking, risk taking, and impulsivity [6]. These personality characteristics correlate significantly with both risky driving behaviour and a higher probability of involvement in road accidents.

As already mentioned, one of the personality characteristics linked to risky driving is the locus of control. The locus of control is a strong person’s conviction in the source of an action or the control of that action [27]. There are two types of locus of control: external and internal. The external locus of control is characterized by a transfer of responsibility for the actions of the individual on other people, luck, chance, or situational factors beyond the control of the self. This can lead to a lack of attention and a lack of precautionary measures when avoiding negative results from a given action. The internal locus of control leads to the adoption of stable actions by the individual, taking responsibility and attempting to avoid negative results from these actions. The locus of control is associated with many attitudes and behaviours related to the driving process, including risky and aggressive driving. The external locus of control is associated with increased involvement in car accidents [28], with driving under the influence of alcohol [29], driving errors [30], and intention of committing violations [31]. The internal locus of control is associated with the use of a safety belt [32], cautious driving behaviour [33], higher vigilance [27], and faster braking when there is a perceived potential danger on the road [34]. Some research indicates that drivers with an increased internal locus of control score higher on risky driving style and drivers with an external locus of control score higher on dissociative, anxious, and distress-reduction styles [35].

Another personality characteristic related to risky driving behaviour is impulsivity. Impulsivity is related to functional impairment in the frontal lobe which was found in personality with affect and interpersonal dysregulation [36,37]. One of the most popular definitions of impulsivity is the predisposition of unplanned reactions as a result of internal and external stimuli without thinking about the negative consequences for the individual and for others [38]. There are two types of impulsivity: functional and dysfunctional [39]. Both lead to fast and error-prone actions but are different in essence. Functional impulsivity expresses a willingness and ability to take risks in situations where this behaviour is appropriate and necessary, while dysfunctional impulsivity expresses the tendency for thoughtlessness and inability to plan and think one’s actions through, leading to negative consequences [40]. Regarding the driving process, impulsivity is essential for traffic safety as it is related to risky behaviour on the road, involvement in road accidents, more driving mistakes, and traffic violations [41]. High-risk drivers have higher scores in both functional impulsivity and dysfunctional impulsivity [40]. It is found that impulsivity is associated with self-reported aberrant driver behaviours and driving anger/aggression, driving under the influence of alcohol, traffic offences, and accidents [42]. According to a study, the dysfunctional impulsivity is significantly positively related to aggressive and ordinary violations, while functional impulsivity was not significantly related to any of these violation types [43]. Also, functional impulsivity is positively, whereas dysfunctional impulsivity is negatively associated with positive driver behaviours [44].

### 1.3. Aim of The Study

On the bases of the analysis of the scientific literature, it can be summarized that drivers prone to risky driving behaviour, different traffic rules violations, and road accident involvement have high levels of anger, aggression, risk taking, impulsivity, social irresponsibility, and sensation seeking [8,9,10,11,21]. According to different studies, other significant characteristics are anxiety, locus of control, and aggressive driving behaviour [21,24,45,46,47,48]. These characteristics are most often associated with risky driving and correlate significantly with the higher probability of participation in road accidents.

It is clear that the driving style can be considered a steady predictor of certain driving behaviour. That is why the main objective of this study is to investigate the interconnection between the driving styles, traffic locus of control, and impulsivity in Bulgarian drivers. It is expected that the driving styles from the maladaptive domains (risky, high-velocity, anxious, dissociative, distress-reduction, and angry styles) will be significantly and positively connected to the external locus of control and the impulsivity, while the driving style from the adaptive domain (patient and careful style) will show a significant and negative connection to these constructs. It is also expected that the patient and careful driving style will be significantly and positively connected to the internal locus of control.

## 2. Materials and Methods

### 2.1. Participants

Four hundred and fifty six Bulgarian drivers from the general population participated in this study. Data collected from October 2018 until December 2018. The age of the participants ranged between 19 and 72 years (M = 37.00; SD = 9.07). According to gender, the sample includes 204 (45%) men and 252 (55%) women. Regarding occupation, the largest percentage of the participants (87%) are from the city of Sofia, and the remaining participants (13%) are from various geographical areas in Bulgaria (Plovdiv, Varna, Pleven, Burgas, Haskovo, etc.). Concerning education, 79.4% of the participants possess a university degree and 20.6% completed high school.

All of the participants owned a driving license, 86.8% of them drive every day or at least two to three times per week, and 13.2% drive two to three times per month or even rarely. Surveyed participants who have registered traffic violations in the past three years are 69.5%. Violations that are most often recorded include speeding, illegal parking, using a phone while driving, driving without a seat belt, and driving after alcohol consumption. The sample also includes 6.1% of surveyed persons who have been deprived of a driving license due to different offenses, most often for speeding, driving after alcohol consumption, and involvement in road accidents. Participants who had caused a road accident are 39%. Victims in a traffic accident caused by another driver are 58.8% of the surveyed persons.

### 2.2. Measures

The Bulgarian version of the Multidimensional driving style inventory was used to assess the driving styles [49]. The MDSI-BG consists of 57 items measuring eight driving styles: Risky, Irrational, Anxious, High velocity, Dissociative, Patient and careful, Angry, and Distress reduction driving styles. The consistency for the whole questionnaire is very high (α = 0.78).

A short version of Traffic locus of control scale [50] developed for Bulgarian context was used to measure the locus of control in driving and the degree to which a driver perceives driving events to be under their own control (internal locus of control) or under the control of other outside factors (external locus of control). The instrument consists of 20 items and the consistency of the whole questionnaire is very high (α = 0.76).

An adapted short version of Dickman’s impulsivity inventory [39] was used to measure the functional and dysfunctional impulsivity in the studied sample. The adapted version consists of 11 items and showed very high consistency of the whole instrument (α = 0.78).

To adopt the instruments to the Bulgarian cultural context as much as possible, some differences and modifications were made. For the purposes of the correct translation into Bulgarian and the development of a suitable method for the Bulgarian sample, the following procedures were performed:

- Right and reverse translation in items formulation;
- Evaluation by experts (three psychologists and one road safety expert);
- Evaluation consistency (approximately 90%).

All of the items are compared to their original versions and edited according to the inconsistencies so as to achieve the highest possible level of correspondence between the original items and their translation into Bulgarian.

Participants also reported their socio-demographic characteristics—age, gender, education, occupation. They were also asked to provide some information regarding their driving experience—years of driving, type of license, how often do they drive, what kind of violations they’ve made in the last three years, caused traffic incidents, etc. Participants included in the study were not paid for their participation.

### 2.3. Procedure

An online version of the questionnaires was prepared to facilitate the process of data collection. A link to the battery was sent to email and social media contacts selected on a random basis (every third of the contact list). Participants were informed that the research is part of a scientific project financed by a program for supporting young scientists in Bulgaria. The response rate was more than 95%. The questionnaire was completed in 20 min on average. Data analysis were performed using SPSS version 25. The statistical methods used for the analysis of the data were Descriptive statistic; and Pearson Correlation.

## 3. Results

Results regarding the interrelations between the driving styles, the traffic locus of control, and the impulsivity are presented in the following subsections. The relation between the impulsivity and the traffic locus of control is also presented.

### 3.1. Driving Styles in Relation to Traffic Locus of Control

The results regarding the relationship between the driving styles and the traffic locus of control are presented in Table 1.

The results show positive correlations between the dissociative and distress-reduction driving styles and the external locus of control. Angry and risky driving styles are negatively associated with the internal locus of control. The patient and careful driving style show a positive correlation with the internal locus of control. Although the values are not high, they are significant. The results didn’t show any correlations between the anxious, high velocity and the irrational driving styles, and the traffic locus of control.

### 3.2. Driving Styles in Relation to Impulsivity

The presented results show significant correlations between all of the driving styles and the Impulsivity. The results are presented in Table 2.

The results show that angry, anxious, dissociative, high-velocity, irrational, distress-reduction, and risky driving styles are significantly positively associated with impulsivity. There is also a significant negative correlation between the patient and careful driving style and impulsivity.

Regarding the functional and the dysfunctional impulsivity, the results are presented in Table 3.

Angry, anxious, high velocity and risky driving styles show significant correlations with both functional and dysfunctional impulsivity. The correlations are positive except the one between the anxious driving style and the functional impulsivity, which is negative. Irrational and distress-reduction driving styles show a significant positive correlation only with the functional impulsivity. Dissociative driving style shows a significant positive correlation only with the dysfunctional impulsivity. The patient careful driving style shows a significant negative correlation with both functional and dysfunctional impulsivity.

## 4. Discussion

Statistically significant correlations are observed between impulsivity, traffic locus of control, and driving styles as it was expected.

Regarding the driving styles and the traffic locus of control, statistically significant positive relationships are observed only with some of the maladaptive driving styles. The angry and risky driving styles are negatively associated with the internal locus of control. That means that if an individual demonstrates an internal locus of control, it is not likely for them to exercise an angry or risky driving style. The angry driving style is characterized by furious outbursts during driving, most often expressed with acts of verbal and instrumental aggression. These reactions are often provoked by external factors, and that is why taking responsibility and attempting to avoid negative results when driving is unlikely. The same concerns the risky style where driving is associated with adrenaline, thrill, and enjoyment of actions that are insecure, dangerous and threatening. Individuals with risky driving style have a sense of complete control over the vehicle and if a road accident occurs, they are likely to accuse external factors rather admit any kind of guilt. This result is logical and expected, knowing that both styles are associated with dangerous driving behaviours [8], which is not typical for internal locus of control where there is a negative association with risky behaviour and sensation seeking in traffic [51]. The dissociative and distress-reduction styles show positive correlations with the external locus of control. That means that the more the external locus of control, the more probability to exercise dissociative or distress-reduction style. The dissociative driving style is characterized by illogical and untypical actions while driving as well as often distractions. Distractions while driving are also typical for the distress-reduction style. It is expected for individuals who seek external factors to relieve the experienced stress to manifest external locus of control in general. This supports suggestions from previous studies where the dissociative style is more typical for people with an external locus of control and may experience greater accident risk, given associations between dissociative style and accident occurrence [9,35]. These results show that individuals who are often distracted while driving are inclined to transfer responsibility for their actions on external factors. As expected, the patient and careful driving style is positively connected with the internal locus of control, characterized by the ability to adopt strategies and stable actions. That is the more internal a participant’s locus of control is, the higher his/her tendency to adopt the patient and careful driving style. This result is logical, because individuals who are careful, concentrate, and patient while driving are more likely to accept and take responsibility if a road accident occurs. These results are partially similar to those obtained in other studies [51].

Analysis of the results shows that despite the low values, there are significant correlations between the driving styles and the impulsivity. The impulsivity as a personality trait is characterized by unplanned reactions without thinking about the negative consequences and is expected to correlate positively with driving styles such as angry, anxious, dissociative, high-velocity, irrational, and risky styles. The highest correlation is between the anxious driving style and impulsivity. That means that the more impulsive the individual is, the more likely it is for them to practice anxious driving style. Although this style is associated with careful and cautious behaviour on road to avoid possible situations of distress and anxiety, it is mainly characterized by a sense of anxiety and nervousness while driving. This may lead to dangerous and risky driving behaviour, which is typical for the maladaptive driving domain. This result is similar to the results demonstrated in other studies, where impulsivity is associated with self-reported aberrant driver behaviours and driving anger, driving under the influence, and traffic offences and accidents [42,43,44]. The other two driving styles from the maladaptive domain associated with impulsivity are the risky and the high-velocity styles. That means that the more impulsive the individual is, the more likely it is for them to exercise risky or high-velocity driving style. These styles often refer to behaviours of seeking sensation and thrill while driving [11]. Typical driving actions for these styles are speeding, racing in cars, passing other cars in no-passing zones, drunk driving, etc. [9]. The results from this research are similar to other studies which discover a connection between the impulsiveness and risky driving [40,42,44]. The next driving style from the maladaptive domain connected to impulsivity is the angry style. That means that the higher the impulsivity, the more likely it is for the individual to adopt angry driving style. The main characteristics of this style refer to expressions of irritation, rage, and hostile attitudes, and acts while driving, mainly manifest with aggressive acts on the road [9]. The results also show that the patient and careful driving style is negatively associated with impulsivity. That means that the lower the impulsivity, the more likely it is for an individual to adopt the patient and careful driving style. This is an expected result, mainly because this style is described with adherence to traffic rules, lack of distraction, readiness to react in any type of road situation, self-control during traffic jams, patience, and good route planning [9,10,11].

Studies investigating functional and dysfunctional impulsivity in the driving context are rather limited and contradictory [42,44]. That is why both functional and dysfunctional dimensions of impulsivity are investigated. Additional analyses are carried out to observe differences between the impulsivity dimensions and the driving styles. On one hand, most of the driving styles from the maladaptive domain are positively associated both with the dysfunctional and the functional impulsivity. That means that no matter which one of the impulsivity dimensions is typical for an individual, it is more likely for them to adopt some of the maladaptive driving styles. Moreover, a driver who adopts some of the maladaptive driving styles can be expected to perform reckless and/or aggressive actions while driving and it is demonstrated that high-risk drivers show higher scores in both functional and dysfunctional impulsivity [40]. On other hand, the patient and careful driving style from the adaptive domain is negatively associated to both functional and dysfunctional impulsivity, therefore if an individual is less impulsive, the probability they are to drive patient and careful is bigger. In general, these results show that the more an individual is impulsive, the more he/she exercises one of the negative driving styles. Conversely, the absence of impulsivity leads to the presence of a positive driving style, which reduces the likelihood of impulsive behaviour on road. These results support the proposition that functional and dysfunctional impulsivity are two distinct yet related constructs stated in other studies [39,44].

## 5. Conclusions

In summary, the results from the conducted study show that if an individual has an external locus of control and/or is impulsive, it is more likely for them to perform a maladaptive driving style, whereas the internal locus of control and the absence of impulsivity lead to a bigger probability for adoption an adaptive driving style.

This research is one of the few contemporary studies in the field of transport psychology in Bulgaria. The study of the driving style as a construct and a key component related to risky behaviour on road provides a good opportunity to learn more about the behaviour of drivers in Bulgaria. Such studies are important in order to increase the level of road safety in the country through policies based on scientific evidence. The real challenge is in understanding the role of the human factor and moreover the consequences of human driving behaviour. Effective countermeasures are needed in order to prevent causation of road accidents. This research is a good base for developing different prevention and intervention programs. Prevention modules revealing the characteristics associated with risky driving and the possible results of such driving can be beneficial to all road users. Intervention programs based on the knowledge gained in this research can also be developed (for example: working with drivers who have different traffic rules violations, drivers who demonstrate a maladaptive driving style, and drivers who are impulsive or transfer the responsibility for their actions on external factors). The study may be also useful in different advertisement and communication campaigns on prevention. This is an opportunity to draw public attention to the importance of the human factor for road safety and a step towards developing a responsible attitude on the subject.

## Figures and Tables

**Table 1:** Correlations between the driving styles and the internal and external traffic locus of control.

		Angry Style	Anxious Style	Dissociative Style	Distress-reduction	High Velocity	Irrational	Patient Careful	Risky Style
**Internal locus of control**	Pearson Correlation	−0.121 **	0.085	0.054	0.052	−0.088	−0.075	0.143 **	−0.180 **
	Sig. (2-tailed)	**0.010**	0.069	0.253	0.267	0.062	0.110	**0.002**	**0.000**
	N	456	456	456	456	456	456	456	456
**External Locus of Control**	Pearson Correlation	0.064	0.029	0.117 *	0.239 **	0.042	−0.033	0.090	−0.029
	Sig. (2-tailed)	0.175	0.537	**0.013**	**0.000**	0.376	0.477	0.056	0.534
	N	456	456	456	456	456	456	456	456

** Correlation is significant at the 0.01 level (2-tailed). * Correlation is significant at the 0.05 level (2-tailed).

**Table 2:** Correlations between the driving styles and impulsivity.

		Angry Style	Anxious Style	Dissociative Style	Distress-reduction	High Velocity	Irrational	Patient Careful	Risky Style
**Impulsivity**	Pearson Correlation	0.234 **	0.293 **	0.176 **	0.140 **	0.251 **	0.170**	−0.257 **	0.262 **
	Sig. (2-tailed)	**0.000**	**0.005**	**0.000**	**0.003**	**0.000**	**0.000**	**0.000**	**0.000**
	N	456	456	456	456	456	456	456	456

** Correlation is significant at the 0.01 level (2-tailed).

**Table 3:** Correlations between the driving styles and the functional and the dysfunctional impulsivity.

		Angry Style	Anxious Style	Dissociative Style	Distress-reduction	High Velocity	Irrational	Patient Careful	Risky Style
**Dysfunctional impulsivity**	Pearson Correlation	0.214 **	0.132 **	0.199 **	0.056	0.200 **	0.041	−0.169 **	0.148 **
	Sig. (2-tailed)	**0.000**	**0.005**	**0.000**	0.231	**0.000**	0.384	**0.000**	**0.002**
	N	456	456	456	456	456	456	456	456
**Functional impulsivity**	Pearson Correlation	0.250 **	−0.204 **	0.057	0.180 **	0.193 **	0.193 **	−0.240 **	0.299 **
	Sig. (2-tailed)	**0.000**	**0.000**	0.227	**0.000**	**0.000**	**0.000**	**0.000**	**0.000**
	N	456	456	456	456	456	456	456	456

** Correlation is significant at the 0.01 level (2-tailed).

## References

[1] Arthur W., Barret G.V., Alexander R.A. (1991). Prediction of Vehicular Accident Involvement: A Meta-Analysis. Hum. Perform..

[2] Parker D., Manstead A.S.R., Semin G.R., Fiedler K. (1996). The social psychology of driver behavior. Applied Social Psychology.

[3] Racheva R., Totkova Z., Shtetinski D., Andreev B. (2019). Validity and Reliability of Methods for Assessing Cognitive Functions in Drivers. Psychol. Res..

[4] Grayson G.B., Maycock G. (1988). Road User Behavior: Theory and Research.

[5] Hennessy D.A., Porter B. (2011). Social, personality, and affective constructs in driving. Handbook of Traffic Psychology.

[6] Totkova Z., Racheva R. (2018). Personality Characteristics Related to Risky Driving Behavior. Psychol. Res..

[7] Totkova Z., Racheva R. (2019). A Multidimensional Method for Assessing Personality Characteristics Related to Risky Driving Behavior. Psychol. Res..

[8] Wang Y., Qu W., Ge Y., Sun X., Zhang K. (2018). Effect of personality traits on driving style: Psychometric adaption of the multidimensional driving style inventory in a Chinese sample. PLoS ONE.

[9] Taubman-Ben-Ari O., Mikulincer M., Gillath O. (2004). The multidimensional driving style inventory—Scale construct and validation. Accid. Anal. Prev..

[10] Poó F., Taubman-Ben-Ari O., Ledesma R., Díaz-Lázaro C. (2013). Reliability and validity of a Spanish-language version of the multidimensional driving style inventory. Transp. Res. Part F Traffic Psychol. Behav..

[11] Holman A., Havârneanu C. (2015). The Romanian version of the multidimensional driving style inventory: Psychometric properties and cultural specificities. Transp. Res. Part F Traffic Psychol. Behav..

[12] Gwyther H., Holland C. (2012). The effect of age, gender and attitudes on self-regulation in driving. Accid. Anal. Prev..

[13] Miller G., Taubman-Ben-Ari O. (2010). Driving styles among young novice drivers-The contribution of parental driving styles and personal characteristics. Accid. Anal. Prev..

[14] Taubman-Ben-Ari O., Skvirsky V. (2016). The multidimensional driving style inventory a decade later: Review of the literature and re-evaluation of the scale. Accid. Anal. Prev..

[15] Poó F.M., Ledesma R.D. (2013). A Study on the Relationship between Personality and Driving Styles. Traffic Int. J. Prev..

[16] Vu H.M., Tran T.T., Vu G.T., Nguyen C.T., Nguyen C.M., Vu L.G., Tran T.H., Tran B.X., Latkin C.A., Ho C.S.H. (2019). Alcohol Use Disorder among Patients Suffered from Road Collisions in a Vietnamese Delta Province. Int. J. Environ. Res. Public Health.

[17] Donovan J.E., Jessor R. (1985). Structure of problem behavior in adolescence and young adulthood. J. Consult. Clin. Psychol..

[18] Taubman-Ben-Ari O., Yehiel D. (2012). Driving styles and their associations with personality and motivation. Accid. Anal. Prev..

[19] Vu H.M., Dang A.K., Tran T.T., Vu G.T., Truong N.T., Nguyen C.T., Doan A.V., Pham K.T.H., Tran T.H., Tran B.X. (2019). Health-Related Quality of Life Profiles among Patients with Different Road Traffic Injuries in an Urban Setting of Vietnam. Int. J. Environ. Res. Public Health.

[20] Goh R.K., Ho R.C., Ng B.Y. (2019). Post-Traumatic Stress Disorder in Road Traffic Accident Survivors—Can We Do More?. Ann. Acad. Med. Singap..

[21] Arnett J.J., Offer D., Fine M.A. (1997). Reckless driving in adolescence: ‘State’ and ‘trait’ factors. Accid. Anal. Prev..

[22] Beirness D.J., Simpson H.M. (1988). Lifestyle correlates of risky driving and accident involvement among youth. Alcohol Drugs Driv..

[23] Beirness D.J., Simpson H.M. (1991). Predicting young driver crash involvement: The role of lifestyle factors. International Symposium New to the Road.

[24] Jonah B.A. (1997). Sensation seeking and risky driving: A review and synthesis of the literature. Accid. Anal. Prev..

[25] Trimpop R.M., Kerr J.H., Kirkcaldy B.D. (1999). Comparing personality constructs of risk-taking behavior. Personal. Individ. Differ..

[26] Zuckerman M., Neeb M. (1980). Demographic influences in sensation seeking and expressions of sensation seeking in religion, smoking, and driving habits. Personal. Individ. Differ..

[27] Rotter J. (1966). Generalized expectancies for internal versus external control of reinforcement. Psychol. Monogr. Gen. Appl..

[28] Lajunen T., Summala H. (1995). Driving experience, personality, and skill and safety-motive dimensions in drivers’ self-assessments. Personal. Individ. Differ..

[29] Cavaiola A., Desordi E. (2000). Locus of Control in Drinking Driving Offenders and Non-Offenders. Alcohol. Treat. Q..

[30] Breckenridge R. (1991). Locus of control and alcohol placebo effects on performance in a driving simulator. Percept. Mot. Ski..

[31] Yagil D. (2006). Reasoned Action and Irrational Motives: A Prediction of Drivers’ Intention to Violate Traffic Laws 1. J. Appl. Soc. Psychol..

[32] Hoyt M.F. (1973). Internal–external control and beliefs about automobile travel. J. Res. Personal..

[33] Montag I., Comrey A.L. (1987). Internality and externality as correlates of involvement in fatal driving accidents. J. Appl. Psychol..

[34] Rudin-Brown C.M., Parker H.A. (2004). Behavioural adaptation to adaptive cruise control (ACC): Implications for preventive strategies. Transp. Res. Part F Traffic Psychol. Behav..

[35] Holland C., Geraghty J., Shah K. (2010). Differential moderating effect of locus of control on effect of driving experience in young male and female drivers. Personal. Individ. Differ..

[36] Husain S.F., Tang T.B., Yu R., Wilson W.T., Bach T., Travis T.Q., Shi-Hui H., Cheryl W.C., Cyrus S.H., Roger C.H. (2020). Cortical haemodynamic response measured by functional near infrared spectroscopy during a verbal fluency task in patients with major depression and borderline personality disorder. EBioMedicine.

[37] Keng S.L., Lee Y., Drabu S., Hong R.Y., Chee C.Y.I., Ho C.S.H., Ho R.C.M. (2019). Construct Validity of the McLean Screening Instrument for Borderline Personality Disorder in Two Singaporean Samples. J. Pers. Disord..

[38] Stanford M.S., Mathias C.W., Dougherty D.M., Lake S.L., Anderson N.E., Patton J.H. (2009). Fifty years of the Barratt Impulsiveness Scale: An update and review. Personal. Individ. Differ..

[39] Dickman S.J. (1990). Functional and dysfunctional impulsivity: Personality and cognitive correlates. J. Personal. Soc. Psychol..

[40] Paaver M., Eensoo D., Pulver A., Harro J. (2006). Adaptive and maladaptive impulsivity, platelet monoamine oxidase (MAO) activity and risk-admitting in different types of risky drivers. Psychopharmacology.

[41] Pearson M., Murphy E., Doane A. (2013). Impulsivity-like traits and risky driving behaviors among college students. Accid. Anal. Prev..

[42] Bıçaksız P., Özkan T. (2016). Impulsivity and driver behaviors, offences and accident involvement: A systematic review. Transp. Res. Part F Psychol. Behav..

[43] Bıçaksız P., Özkan T. (2016). Developing the impulsive driver behavior scale. Transp. Res. Part F Psychol. Behav..

[44] Bıçaksız P., Öztürk İ., Özkan T. (2019). The differential associations of functional and dysfunctional impulsivity with driving style: A simulator study. Transp. Res. Part F Traffic Psychol. Behav..

[45] Ulleberg P. (2002). Personality subtypes of young drivers. Relationship to risk-taking preferences, accident involvement, and response to traffic safety campaign. Transp. Res. Part F Traffic Psychol. Behav..

[46] Jonah B.A., Thiessen R., Au-Yeung E. (2001). Sensation seeking, risky driving and behavioral adaptation. Accid. Anal. Prev..

[47] Rosenbloom T. (2003). Sensation seeking and risk taking in mortality salience. Personal. Individ. Differ..

[48] Dula C.S., Ballard M.E. (2003). Correlates of aggressive, risky, and emotional driving. J. Appl. Soc. Psychol..

[49] Totkova Z., Racheva R. (2019). The Bulgarian version of Multidimensional driving style inventory: Psychometric properties. Behav. Sci..

[50] Özkan T., Lajunen T. (2005). Multidimensional Traffic Locus of Control Scale (T-LOC): Factor structure and relationship to risky driving. Personal. Individ. Differ..

[51] Măirean C., Havârneanu G., Popușoi S., Havârneanu C. (2017). Traffic locus of control scale—Romanian version: Psychometric properties and relations to the driver’s personality, risk perception, and driving behavior. Transp. Res. Part. F Traffic Psychol. Behav..

